# Modulation of fatty acid profiles and turnover dynamics in jellyfish polyps through copepod diets: Insights into trophic interactions and nutrient flux

**DOI:** 10.1002/ece3.70332

**Published:** 2024-10-22

**Authors:** Xupeng Chi, Fang Zhang, Song Sun

**Affiliations:** ^1^ Key Laboratory of Marine Ecology and Environmental Sciences, Institute of Oceanology Chinese Academy of Sciences Qingdao China; ^2^ Laboratory for Marine Ecology and Environmental Science, Laoshan Laboratory Qingdao China; ^3^ College of Marine Science University of Chinese Academy of Sciences Qingdao China

**Keywords:** bioconversion, copepod, fatty acid, food quality, jellyfish, turnover

## Abstract

Fatty acids (FAs) are vital biomolecules crucial for determining food quality for higher trophic levels. To investigate FA transfer and turnover time in predators, we conducted a diet switch experiment using jellyfish polyps. These polyps were fed food sources including *Artemia sinica* nauplii and FA‐manipulated copepod *Pseudodiaptomus annandalei*, maintained on distinct algal diets with varied FA compositions. Our findings reveal that copepods may have a strong potential to synthesize long‐chain polyunsaturated FA to maintain biochemical homeostasis when consuming low‐quality food. Consequently, the species‐specific fatty acid composition within plankton, combined with effects of seasonal environmental fluctuations and climate change, leads to changes in the FA composition of foundational food web components. These alterations create a complex “nutrient black box” effect as they propagate up trophic levels. Our study shows that jellyfish polyps fail to accumulate EPA and DHA but display high levels of ARA compared to their zooplankton and phytoplankton food sources, suggesting a potential association with dietary EPA and DHA through an unidentified pathway. Certain FA components indicate variations in the turnover time when polyps undergo a dietary shift. Understanding the trajectory of FA metabolism across the “phytoplankton–zooplankton” interface, along with its turnover time, provides crucial insights for modeling diet estimation of components within food webs.

## Graphical abstract

The copepod exhibits a strong inclination toward synthesizing long‐chain polyunsaturated FA to maintain biochemical homeostasis when consuming low‐quality food. Jellyfish polyps fail to accumulate EPA and DHA but display high levels of ARA compared to their zooplankton and phytoplankton food sources, suggesting a potential association with dietary EPA and DHA through an unidentified pathway. Certain FA components suggest a turnover time of approximately 19 days when polyps undergo a dietary shift.

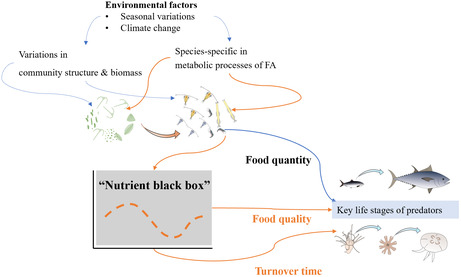

## 1 INTRODUCTION

Understanding matter cycling and energy flow pathways within food webs is paramount for gaining insights into ecosystem functioning. Essential biomolecules synthesized by phytoplankton and zooplankton have been identified as key determinations of food quality for predators, thereby exerting “bottom‐up” control over higher trophic levels by regulating their metabolism, growth, and reproduction (Singh et al., 2023; Vesterinen et al., 2022). Despite the documented importance of essential or semi‐essential biomolecules in food webs, a comprehensive understanding of their transfer and turnover along the food chain remains incomplete.

Among the essential biomolecules, fatty acids (FAs) play crucial roles in organisms' energy storage, membrane fluidity, and serve as precursors for tissue hormones (Müller‐Navarra, 2008), thereby regulating organisms' fitness and reflecting lipid‐based energy flow within the food web. Long‐chain polyunsaturated fatty acids (LC‐PUFA), such as EPA (C20:5n3) and DHA (C22:6n3), are particularly critical for zooplankton egg production and hatchment (Arendt et al., 2004), supplementation of fish stocks (Singh et al., 2023), and human health (Pereira et al., 2003). Organisms possess enzymes facilitating the de novo production of LC‐PUFA, a process reliant on the coexistence of both Δ12 and Δ15 desaturases (Kabeya et al., 2018). In marine pelagic food webs, LC‐PUFAs are typically biosynthesized de novo by phytoplankton, heterotrophic protists, and bacteria (Jonasdottir, 2019). Even though the elongates and desaturases have been increasingly described in many organisms, most zooplankton and other heterotrophic predators only have limited ability to synthesize PUFA de novo due to the absence of necessary elongating and desaturating enzymes (Monroig & Kabeya, 2018; Pereira et al., 2003). Consequently, they rely on trophic upgrading of dietary provision to fulfill their own fitness requirements and to supply trophic needs to predators.

Under field conditions, interpreting the FA composition and transfer process of zooplankton, which determines the food quality for predators, presents a multifaceted challenge. On one hand, the foundational component of the food web, namely phytoplankton, exhibits highly species specificity and environmentally dependence in PUFA biosynthesis. For instance, while most green algae are abundant in ALA (C18:3n3), diatoms and cryptophytes are generally rich in EPA, and dinoflagellates and haptophytes are abundant in DHA (Ruess & Müller‐Navarra, 2019). Furthermore, seasonal environmental fluctuations not only influence variations in biomass and community structure within the basal food web (Sommer et al., 2012) but also contribute to intra‐specific differences in their FA composition over time. Consequently, the food supply of predators in the field undergoes shifts in both quantity and quality. On the other hand, owing to the intricate processes of catabolism and anabolism in zooplankton, FA profiles undergo modifications as they ascend the food chain, rendering the quantitative tracking FA transfer processes challenging. Notably, prior investigations have demonstrated the presence of relevant enzymes in several invertebrates for PUFA production (Kabeya et al., 2018).

In addition to the transfer process, the timely delivery of sufficient high‐quality food to predators is also essential to support their physiological activities during critical life stages. In this study, the turnover time is defined as the period from when a predator encounters a new food source until it reaches a new biochemical homeostasis. It reflects how long it takes for a predator to incorporate and stabilize the new fatty acid profile after a dietary change, rather than merely the time required for digestion and assimilation. This concept provides a proxy for understanding how long a biomolecule remains in an organism before being metabolized and expelled. The synchronization between food availability and physiological requirements may significantly influence the survival of larvae and consequently impact the population dynamics of predators such as fish or jellyfish (Ferreira et al., 2023). While previous hypotheses such as the “match & mismatch” hypothesis have predominantly focus on food quantity (Asch et al., 2019; Gotceitas et al., 1996), the significance of food quality has been overlooked. Therefore, elucidating the process of FA transfer across the “phytoplankton–zooplankton” bio‐interface is crucial for comprehending biochemical dynamics along the food chain and ensuring authentic food provision for higher trophic levels.

The bloom‐forming moon jellyfish, *Aurelia* spp., exhibit a metagenic life cycle (Arai, 2012), with population outbreaks reported in coastal areas, leading to ecological and socio‐economic challenges (Duarte et al., 2014; Edelist et al., 2021; Stoltenberg et al., 2021). Food conditions, encompassing both quantity and quality, significantly influence the asexual reproduction of their polyp larval stage (Chi et al., 2018, 2019). Consequently, the transfer of FA along the phytoplankton–zooplankton food chain and the turnover time are intricately link to the energetic adaptation and life history strategies of jellyfish polyps. Thus, this study aimed to investigate the FA composition variations across trophic levels, as well as the turnover time of FA transfer to jellyfish polyps within the context of a diet switch experiment.

## 2 MATERIALS AND METHODS

### 2.1 Experimental design and sampling

The moon jellyfish (*Aurelia coerulea*) polyps utilized in this experiment were obtained from permanent laboratory cultures originating from Jiaozhou Bay (120°18′56″ E, 36°3′50″ N), China. Prior to the commencement of the experiment, the polyps were maintained at room temperature and fed with *Artemia sinica* nauplii twice a week. To prepare for the experiment, the polyps were carefully detached from their substrates using forceps and individually transferred into each well (with a growth area: 8.87 cm^2^, volume: 4 mL) of 6‐well polycarbonate culture plates. Each culture unit was filled with filtered (20 μm) ambient seawater and maintained at a constant temperature of 20°C (±0.5°C) with a climate chamber. To facilitate the attachment of polyps to the culture units, they were left undisturbed without food or water exchange for the initial 7‐day acclimation period preceding the start of the experiments. Throughout both the acclimation period and the experiment itself, the polyps were maintained under dark conditions to prevent light from affecting their asexual reproduction and avoid potential biofilm formation by other microorganisms.

**FIGURE 1:**
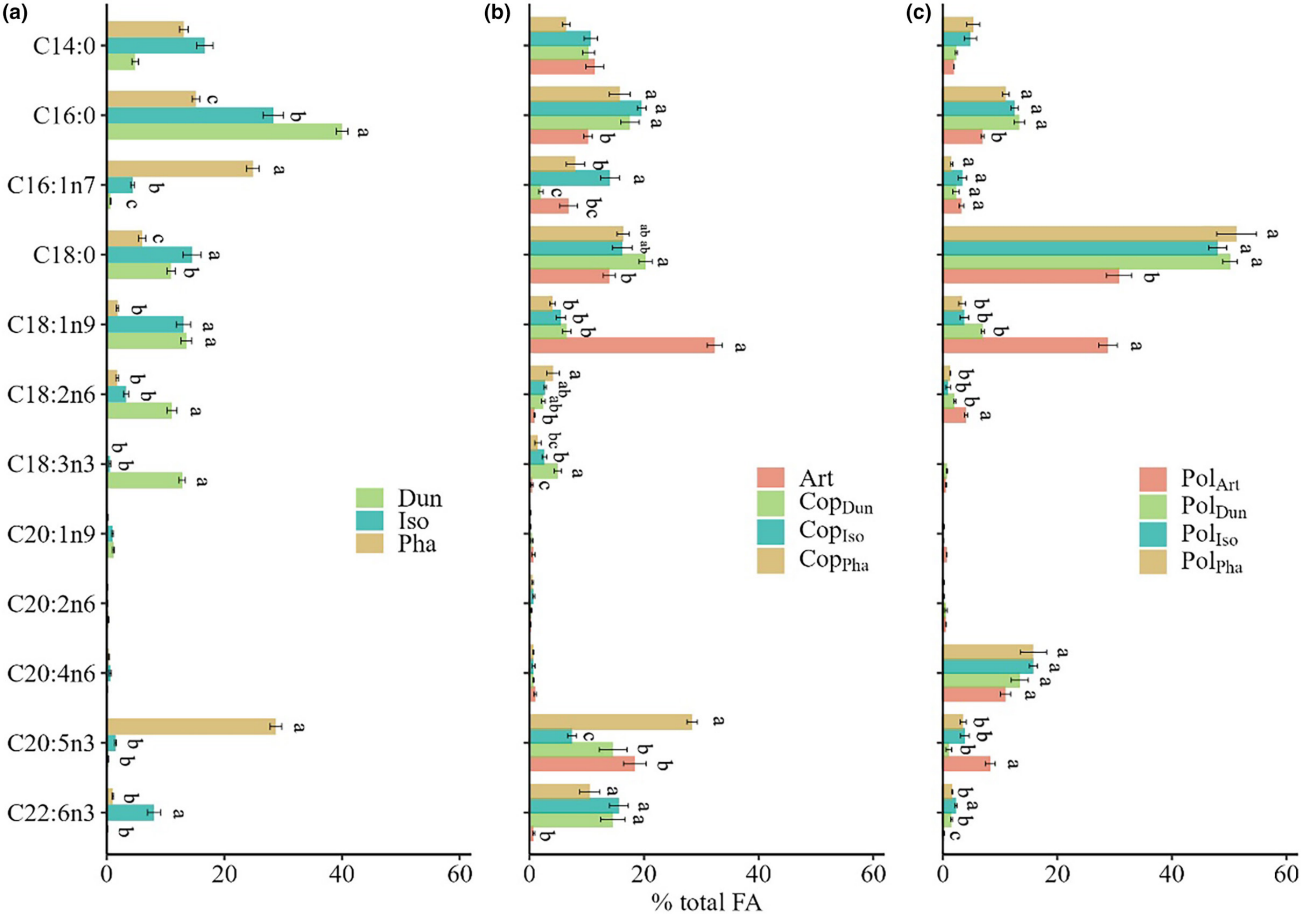
Fatty acid composition of phytoplankton (a), zooplankton (b), and jellyfish polyps (c), with error bars indicating standard errors.

*Artemia sinica* nauplii and the calanoid copepod *Pseudodiaptomus annandalei* served as food sources for the polyps in this experiment. The *A. sinica* nauplii were hatched from eggs originally obtained from a salt lake located in Tibet, China. The copepod *P. annandalei* has been maintained in long‐term culture and were originally isolated from natural coastal waters. For this experiment, they were nourished with three distinct phytoplankton species which were characterized by different FA compositions, namely *Phaeodactylum tricornutum* (referred to as Pha), *Isochrysis galbana* (referred to as Iso), and *Dunaliella salina* (referred to as Dun), respectively (Figure 1). These phytoplankton species were cultured in standard f/2 medium (Guillard, 1975; Guillard & Ryther, 1962). To manipulate the varied food quality of copepods as prey for polyps, they were separately cultured in dedicated tanks (1200 L) for more than 30 days preceding the experiment. During this period, copepods were provided daily with an excess of each of the three phytoplankton species. Subsequently, copepods were collected using a 150‐μm sieve once they attained a stable FA composition, following which they were utilized to feed the polyps. Consequently, four types of zooplankton were provided as food for the polyps in this experiment: *A. sinica* nauplii (referred to as Art), copepod fed with Pha (referred to as Cop_Pha_), copepod fed with Iso (referred to as Cop_Iso_), and copepod fed with Dun (referred to as Cop_Dun_). These treatments for polyps were denoted as Pol_Art_, Pol_Pha_, Pol_Iso_, and Pol_Dun_, respectively. Each treatment contains 100 experimental units, thus, resulting in a total of 400 units. After all polyps were attached to the plates, they were overfed with respective food sources, making the first day of the feeding regime as D01. Any remaining food in each unit was removed during water changes. Throughout the experiment, newly formed buds resulting from asexual reproduction were retained within the same experimental units and subsequently collected alongside the mother polyps for FA analysis.

Polyps from the different treatments were collected every three days for FA analysis. For each sample, polyps were detached from three randomly selected experimental units within each treatment and combined to form three replicates for each of the four food treatments on every sampling day. In order to monitor the consistency of FA composition in phytoplankton and zooplankton, three samples of phytoplankton and zooplankton were collected for FA analysis every 5 days. To prepare the FA samples, approximately 5 mL of each alga species was filtered using GF/F filters (pore diameter = 0.7 μm), while zooplankton from each treatment was stored in 2‐mL tubes. All collected samples were subsequently stored at −80°C prior to FA analysis. The duration of the experiment spanned 31 days.

### 2.2 Fatty acid analysis

All samples of phytoplankton, zooplankton, and polyps underwent lyophilization and homogenization prior to FA composition analyses. For each zooplankton and polyp sample, approximately 1.25 mg of dry weight of homogenized powders was used for lipid extraction. Lipids were extracted at −20°C using a solvent mixture consisting of chloroform, dichloromethane, and methanol in a 1:1:1volume ratio and maintained for a duration of 12 h. The resulting liquid mixture was subsequently separated by the addition of a 1 mol L^−1^ potassium chloride solution in a separating funnel, with the lower liquid phase collected. To this collected phase, 100 μL of toluene and 200 μL of acidified methanol were added for esterification, followed by incubation at 50°C for 12 h. The FA methyl esters (FAMEs) thus produced were purified using a 5% sodium chloride solution and dried using a constant flow of nitrogen gas. Finally, the extracted FAMEs were redissolved in 100 μL of n‐hexane for subsequent analysis. FAMEs were analyzed using a gas chromatograph–mass spectrometer (GC‐MS; Thermo Fisher Scientific), with helium employed as the carrier gas. An external standard, Supelco‐37 FAME mixture (Sigma‐Aldrich), and the internal standard C19:0 (Merck) were utilized for peak identification and quantification based on peak areas.

### 2.3 Statistical analysis

FA results were expressed as a percentage of total FA (% total FA) to interpret dietary differences between treatments and trophic levels (Happel et al., 2017). All detected fatty acids (FAs) were included in the calculation of total FA. However, in subsequent statistical analyses, individual FAs that constituted less than 0.2% of the total FA in most treatment groups were excluded to ensure data robustness and to focus on the most relevant FAs for the study. To assess differences among the FA composition of phytoplankton, zooplankton, and polyp treatments, respectively, a permutational multivariate analysis of variance (PERMANOVA) with Bray–Curtis similarity matrix was conducted. Principal component analysis (PCA) was subsequently employed to visually depict the distinctions between treatments. Differences in specific FA components among food quality treatments were evaluated using one‐way analysis of variance (ANOVA) followed by Tukey's highly significant difference (Tukey HSD) test. Generalized linear models (GLMs) were utilized to analyze how FA components of different treatments varied over time. The calibration coefficients (CCs) as the ratio of predator and prey FA were calculated to trace trophic pathways across trophic levels (Schaub et al., 2021).

A first‐order exponential decay model was utilized to quantify the turnover time of FA (Martínez del Rio & Anderson‐Sprecher, 2008). The data were fitted using the nonlinear least squares regressions. The model describes the FA composition as a function of time, with the following formula:

$$X_{t}=X_{\infty }-\left(X_{\infty }-X_{0}\right)e^{-\mathrm{\lambda t}}$$

where $X_{t}$ is the fatty acid composition at time t, $X_{\infty }$ represents the asymptotic composition, $X_{0}$ is the initial composition, andλ is the turnover rate constant. Initial estimates for the parameters were derived from the experimental data. The fitted model parameters provided the turnover rate constant (λ), which was used to calculate the half‐life (t_1/2_) using the following formulas: t_1/2_ = ln(2)/λ. The model fitting was performed using the “nlsLM” function from the “minpack.lm” package in R. All statistical tests, data explorations, and data visualization procedures were conducted using the R software (R Core Team, 2021).

## 3 RESULTS

### 3.1 Fatty acid composition in different trophic levels

FA composition was significantly different among the three alga diets (PERMANOVA, df = 2, *F* = 155.42, *R* ^2^ = .86, *p* < .01). As depicted in Figure 1, Dun exhibited a higher content of ALA (12.84 ± 0.55%, mean ± se and thereafter, *F*(2, 51) = 495.00, *p* < .001), Iso contained a greater proportion of DHA (8.03 ± 1.11%, *F*(2, 51) = 44.99, *p* < .001), and Pha showcased a higher level of EPA (28.76 ± 1.00%, *F*(2, 51) = 769.7, p < .001). Particularly noteworthy is the deficiency of C20 PUFA in Dun.

In this experiment, the FA composition across the four zooplankton treatments manifested significant distinctions (PERMANOVA, df = 3, *F* = 23.41, *R* ^2^ = .78, *p* < .01). A prominent contrast between Art and the three copepod treatments was the absence of DHA (0.73 ± 0.16%, *F*(3, 20) = 17.52, *p* < .001), albeit with a notable abundance of C18:1n9 (32.35 ± 1.35%, *F*(3, 20) = 219.70, *p* < .001). Conversely, the three copepod treatments had relatively elevated levels of EPA and DHA. Although Art displayed a deficient of DHA, it had second‐highest EPA level (18.38 ± 1.97%).

The polyps had particularly high levels of three FA components: C16:0, C18:0, and ARA (C20:4n6). In comparison with polyps fed with copepods, those fed with Art (Pol_Art_) demonstrated diminished levels of these three components, while Pol_Art_ displayed a significantly elevated content of C18:1n9 (28.85 ± 1.61%, *F*(3, 8) = 163.80, *p* < .001) and a relatively high EPA content (8.31 ± 0.87%, *F*(3, 8) = 19.24, *p* < .001), albeit lacking DHA (0.26 ± 0.04%, *F*(3, 8) = 35.68, *p* < .001). The FA composition among polyps fed with the three copepod treatments exhibited less variation compared to their original Art food source, with generally lower accumulation of EPA and DHA.

### 3.2 Tracking FA components across different trophic levels

Following the trophic pathway from phytoplankton to zooplankton and onward to polyps, a discernible trend emerges in the FA composition. Specifically, the content of short‐chain saturated FA C14:0 and C16:0 exhibits a decline, whereas the content of C18:0 increased. Notably, the abundance of C18:0 in polyps fed with copepods is nearly fourfold higher than that in phytoplankton (Figure 1). Nonetheless, distinctive variations are observed in the levels of PUFA. For instance, the relatively elevated levels of ALA in Dun, EPA in Pha, and DHA in Iso are reflected in the corresponding copepod treatments. Similarly, the heightened levels of C18:1n9 and EPA in Art, along with DHA in the copepod food source, contributed to the analogous levels observed in polyps. The absence of DHA in Art is likewise mirrored in Pol_Art_.

**FIGURE 2:**
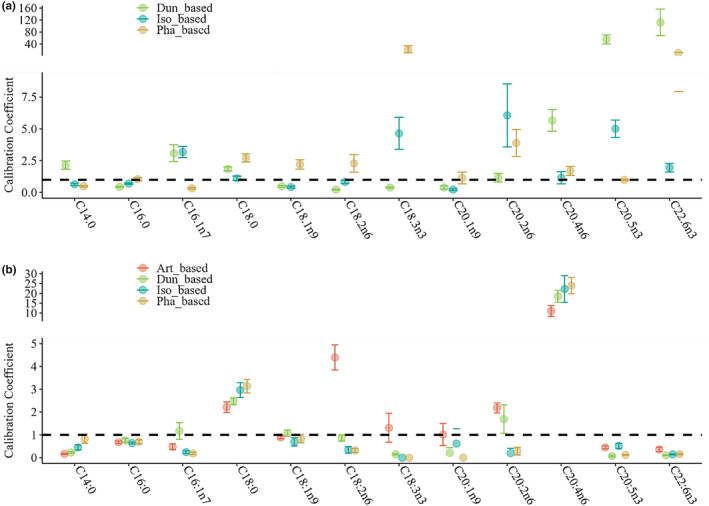
Calibration coefficient estimates of fatty acid across trophic levels: From phytoplankton to zooplankton (a) and from zooplankton to jellyfish polyps (b).

Furthermore, in addition to FA accumulation, FA modification is observed across trophic levels. The most notable modification of FA composition is observed in copepods fed with Dun. Despite Dun lack of EPA and DHA, Cop_Dun_ exhibits high levels of these two components, with CCs of 55.55 ± 15.11 and 112.11 ± 44.47, respectively (Figure 2). Similarly, although ARA is deficient in both phytoplankton and zooplankton food sources, it is abundant in polyps across all treatments. The CCs of ARA from zooplankton to polyps are CC_Art_ = 11.05 ± 2.77, CC_Dun_ = 18.52 ± 3.0, CC_Pha_ = 23.94 ± 4.12, and CC_Iso_ = 22.18 ± 6.85, respectively.

**FIGURE 3:**
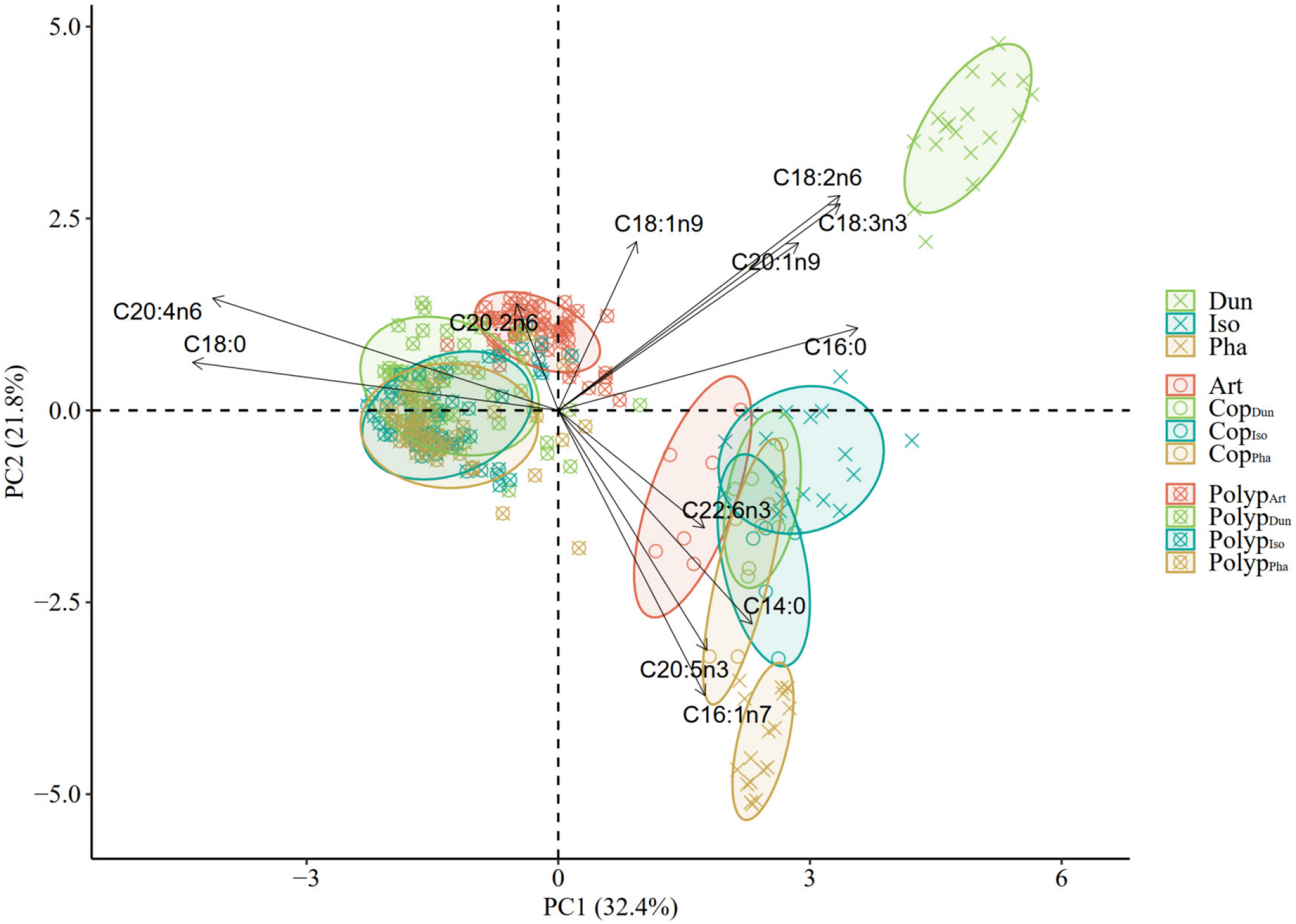
Principal component analysis (PCA) illustrating the FA variations across treatments and trophic levels (ellipse confidence level = 0.75).

The patterns of FA composition across different treatments and trophic levels are further supported by PCA results (Figure 3). PC1 and PC2 collectively explain 54.2% of the total variables. Notably, the substantial distances between phytoplankton ellipses underscore their dissimilarity, with ALA (C18:3n3) serving as a distinctive biomarker for Dun, DHA (C22:6n3) for Iso, and EPA for Pha. Copepod treatments cluster together owing to their shared high DHA content. Overlapping ellipses of polyps fed with copepods arise due to their shared high levels of ARA (C20:4n6). Art and Polyp_Art_ are distinctly separated from zooplankton and polyps, respectively, attributable to the heightened levels of C18:1n9.

### 3.3 Turnover time of zooplankton FA to polyps

**FIGURE 4:**
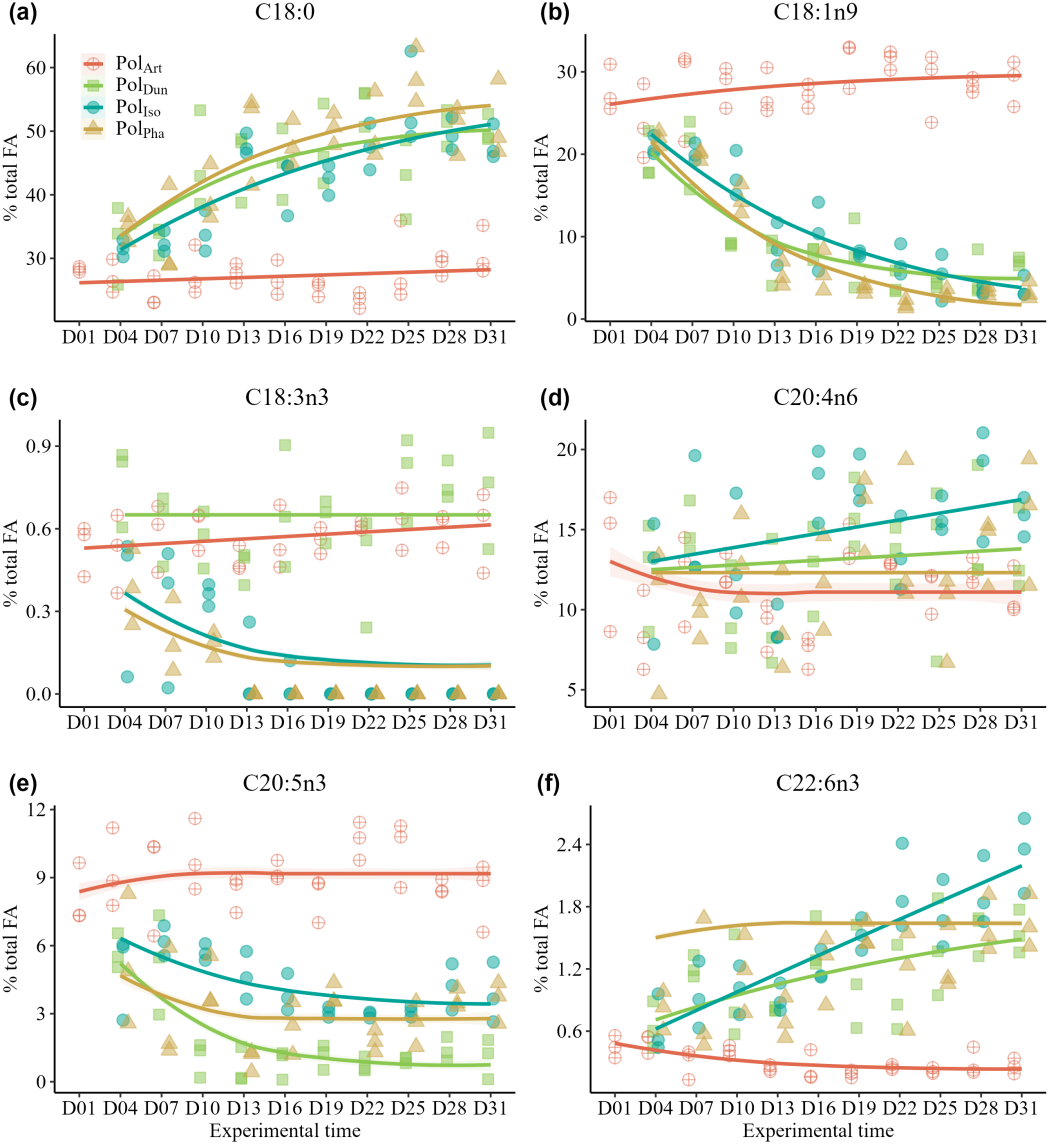
Time series analysis of variations in fatty acid component content upon the transition of polyp diet from *Artemia* to copepods. The trend lines are fitted using nonlinear least squares regressions, and the shaded regions indicated the standard errors.

The FA components of Pol_Art_ remained relatively stable through the duration of the experiment (Figure 4, GLM, df = 10, χ^2^ = 0.002, *p* = .97). However, upon transitioning the food supply from Art to copepods, the FA composition of the polyps exhibited component‐specific variations over time. Notably, there were observed increases in C18:0 (GLM, df = 9, χ^2^ = 0.41, *p* < .001) and DHA (GLM, df = 9, χ^2^ = 0.001, *p* < .001), alongside decreases in C18:1n9 (GLM, df = 9, χ^2^ = 0.34, *p* < .001) and EPA (GLM, df = 9, χ^2^ = 0.01, *p* < .001). Although the content of ARA was initially high across all polyp treatments, it exhibited a marginally significant increase upon switching to a copepod diet (GLM, df = 3, χ^2^ = 0.02, *p* = .09). Overall, the FA composition of polyps fed with copepods appeared to stabilize into a new biochemical equilibrium for some biomarkers such as C18:1n9, C18:3n3, and C20:5n3, with variations in their half‐lives. The half‐life of C18:1n9 in Pol_Dun_, Pol_Iso_, and Pol_Pha_ is 5.38, 10.34, and 7.09 days, respectively. The half‐life of C18:3n3 in Pol_Iso_ and Pol_Pha_ is 4.27 and 3.36 days, respectively. For C20:5n3, the half‐life in Pol_Dun_, Pol_Iso_, and Pol_Pha_ is 4.07, 5.94, and 2.18 days, respectively. However, the variation trends of components such as C18:0, C20:4n6, and C22:6n3 did not reach equilibrium in the polyps fed with copepod treatments.

## 4 DISCUSSION

Investigating the transfer process and turnover dynamics of essential biomolecules across various trophic levels is imperative for comprehending material cycling and energy flux within food webs. In the present study, newly hatched *Artemia* nauplii and biochemically modified copepods were utilized as dietary sources to mimic zooplankton scenarios featuring inter‐ and intra‐specific FA supply supplies. This approach aimed to delineate the transformation mechanisms and turnover kinetics of FA within polyps, thereby furnishing theoretical insights conducive to predicting the population dynamics of jellyfish polyps.

### 4.1 Biochemical adaptations of zooplankton

*Artemia* spp., conventionally employed as a common experimental and aquaculture feed, has been historically perceived as nutritionally inadequate due to its limited content of LC‐PUFA (Navarro et al. 1992; Tizol‐Correa et al., 2006). However, our findings reveal that Art harbors a relatively diminished level of ALA but substantially elevated levels of EPA, constituting approximately 20% of the total FA pool. This underscores the importance of comprehensively discerning the specific strains and nutritional profiles when utilizing *Artemia* spp. as a primary dietary source for laboratory‐reared organisms, as these factors are crucial for accurately interpreting experimental results.

Copepods, as ubiquitous members of zooplankton communities, occupy pivotal trophic niches facilitating the transfer of energy and essential biomolecules synthesized by phytoplankton to higher trophic levels. Our investigations elucidate both the accrual and modification patterns of FA components across the “phytoplankton–zooplankton bio‐interface.” Specifically, the copepod species *P. annandalei* demonstrates not only the capacity to accumulate dietary EPA and DHA but also the potential to biosynthesize these LC‐PUFA from ALA through elongation and desaturation pathways when these constituents are deficient in its diet. Remarkably, high proportions of EPA and DHA accumulated in Cop_Iso_ and Cop_Pha_, respectively, underscore the source of dietary FA components. This adaptive mechanism is further supported by the substantial decrease in ALA and concurrent increase in EPA and DHA observed in Cop_Dun_ relative to their dietary source Dun. Similar processes have been corroborated in other copepod species through radioactive labeling methodologies (Moreno et al., 1979). Although calanoid copepods cannot synthesize PUFA de novo, our findings suggest their potential proficiency in elongating LC‐PUFA from dietary FA precursors. This adaptive strategy ensures the fulfillment of copepods' physiological requirements for LC‐PUFAs through a combination of dietary uptake and endogenous biosynthesis, thereby conferring benefits not only to their own physiological needs but also to predators occupying higher trophic levels. However, further studies using labeling approaches are needed to specifically investigate the biosynthesis progress of LC‐PUFA in copepods.

### 4.2 FA transfer from zooplankton to polyps

Evidently, the levels of saturated FAs C14:0 and C16:0 declined along of the phytoplankton–zooplankton–polyp trophic chain, while the abundance of C18:0 notably increased in polyps. This observation suggests a propensity for polyps to prioritize the synthesis of long‐chain saturated FA for energy storage (Kelly & Scheibling, 2012). Notably, the prevalent presence of the LC‐PUFA EPA in Art was faithfully reflected in polyps. Furthermore, the consistent levels of C18:1n9 from Art to Pol_Art_ indicate that polyps refrain from further modifying C18:1n9 into additional LC‐PUFA, thereby rendering it a suitable biomarker for tracking FA variations when polyps undergo dietary shifts.

Of particular interest is the discrepancy observed in DHA content, which was abundant in all copepod treatments but conspicuously limited in the corresponding polyps. Conversely, ARA exhibited remarkably elevated levels in polyps, despite its initial absence absent in both phytoplankton and zooplankton. Remarkably, ARA levels also surged in Pol_Art_, notwithstanding its absence in Art. This outcome aligns with findings from the prior research (Chi et al., 2018). When polyps were fed Art, rich in ALA yet deficient in EPA and DHA, the resultant polyps harbored minimal ARA levels. Conversely, when copepods, abundant in EPA and DHA, served as dietary source, polyps exhibited heightened ARA levels. From the current widely accepted biosynthesis pathway of ARA, which has to desaturate from a series of “n‐6” pathway, that is, C18:1n9 → C18:2n6 → C18:3n6 → C20:3n6 → C20:4n6 (ARA), or the other way, C18:1n9 → C18:2n6 → C20:2n6 → C20:3n6 → C20:4n6 (ARA). From the result of this experiment, the “n‐6” precursors were poor in the food source. Although the content of C18:1n9 was high in Art, it accumulated almost to a similar level in Pol_Art_, with no evidence of further modification. This observation suggests that jellyfish polyps actively contribute to endogenous ARA production. Interestingly, the elevated ARA content compared to the ambient zooplankton also occurs in medusae (Schaub et al., 2021; Stenvers et al., 2020). Notably, the significant decrease in EPA and DHA levels in polyps implies a probable association between ARA accumulation and dietary EPA and DHA, prompting speculation that jellyfish polyps may biosynthesize ARA from these precursors through an unidentified mechanism. Noteworthy is the retroconversion of ARA from DHA documented in freshwater zooplankton via stable isotope labeling techniques (Strandberg et al., 2014). Additionally, similar to other cnidarian organisms, coral polyps synthesize C20‐22 n‐6 PUFA, facilitating growth and reproduction (Imbs et al., 2010). While microbial contributions cannot be entirely ignored, the preference for ARA through accumulation or modification may exist in jellyfish or even across cnidarian taxa. Given the intricate life history strategy of jellyfish, further investigations are warranted to ascertain whether elevated ARA levels in polyps are linked to growth and asexual reproduction processes, potentially contributing to population outbreaks. Nonetheless, C18:1n9 and ARA emerge as promising biomarkers for exploring trophic relationships between polyps and their predators in natural habitats.

### 4.3 Turnover time

Generally, the turnover time of biochemical composition in zooplankton is species‐specific and contingent upon the characteristics of the ingested food, alongside environmental factors (Azani et al., 2021; Boissonnot et al., 2016; Grosse et al., 2019). Prior investigations have indicated that the turnover time of FA in copepods ranges from approximately 12–14 days (Boissonnot, 2017). According to the findings of this study, when the food source transitioned from *Artemia* nauplii to various copepod treatments, the time required for specific FA components in polyps to stabilize varied. Thus, when food quality changes at the base of the food web, it is estimated that specific FA components require approximately one month to integrate into the polyp's own tissue, supporting their somatic growth and reproduction.

Most scyphozoan jellyfish exhibit a complex and plastic life history strategy. Temperature and food conditions have been identified as the two most important factors influencing the life history strategy and population dynamics of jellyfish (Lucas et al., 2012). In the field, environmental factors and food conditions may undergo significant changes within one month. The degree of temporal synchronization between the food supply available to polyps and their physiological regulation, influenced by environmental factors, directly impacts the growth and asexual reproduction of polyps, consequently affecting their population size and the abundance of jellyfish in subsequent generations.

Prior investigations have confirmed that the timely provision of sufficient food during the breeding season determines the survival of larval stages of organisms and subsequently influences their population dynamics (Cushing, 1969; Ferreira et al., 2023; Raubenheimer et al., 2012). The data on FA turnover time are indispensable for predicting the population dynamics of each life stage of predators. Furthermore, variations of environmental factors under scenarios of global change affect food webs by altering the inputs and transfers of essential biomolecules (Ruess & Müller‐Navarra, 2019). Consequently, investigating the diverse and intricate life history strategies of zooplankton under climate variability and its impact on predator fitness poses a long‐term challenge.

### 4.4 Ecological implications

Environmental factors exhibit significant seasonal variability and are subject to long‐term alterations driven by climate change. These fluctuations profoundly affect aquatic ecosystems, leading to changes in the community structure and biomass of both phytoplankton and zooplankton. Variations in FA composition among these organisms can be attributed to multiple factors. On the one hand, these variations arise from inter‐species differences in metabolic processes, such as biosynthesis, catabolism, anabolism, and selective uptake (Monroig et al., 2013). Additionally, seasonal fluctuations in FA composition among both phytoplankton and zooplankton are notably influenced by environmental parameters such as temperature and light (McLaskey et al., 2022; Nielsen et al., 2023; Wang et al., 2022).

**FIGURE 5:**
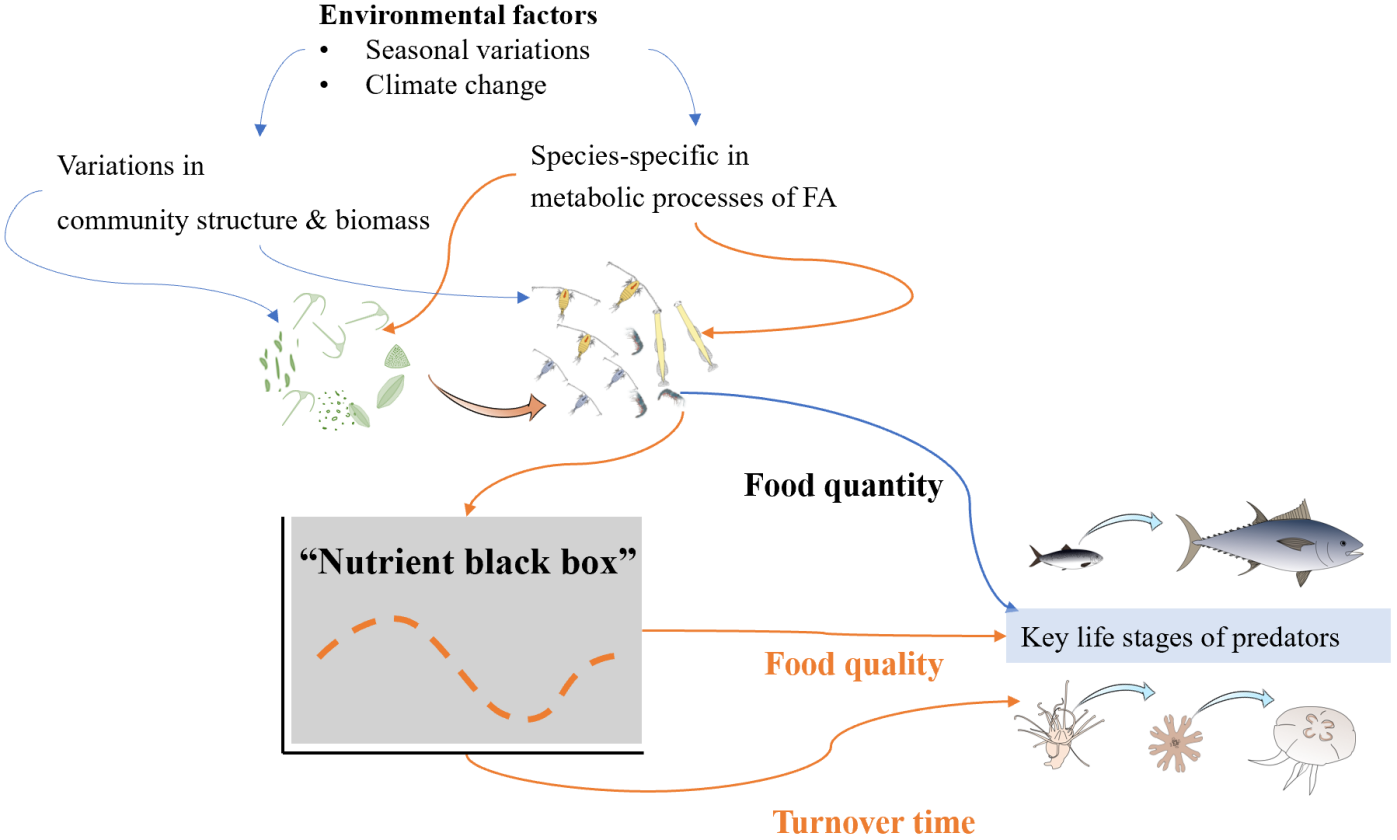
The diagram of “nutrient black box” in marine food webs with emphasis on food quality requirements in the key life stages of predators.

Although the primary focus of this study is the transfer of FA through trophic levels, attention is also given to the fluctuations of other biochemical molecules, such as amino acids, sterols, and vitamins. These variations in biochemical factors contribute to the formation of a complex “nutrient black box” as biomass and energy are transferred up the trophic levels, ultimately impacting the fitness of organisms (Figure 5). Therefore, the ecological inquiry into trophic relationships within food webs extends beyond merely delineating “who eats whom by how much.” It also involves unraveling the intricate dynamic of the “nutrient black box” during pivotal life stages of predators, which inherently shape their population dynamics.

Moreover, the duration required by predators to achieve a new equilibrium state of FA composition following a dietary shift reflects their sensitivity to alterations in food sources. The temporal alignment between the availability of sufficient high‐quality food and the physiological demands of predators is crucial in determining their overall fitness. Alongside FA turnover, the quantity and quality of available food sources are pivotal in shaping the population dynamics of higher trophic levels, including predators such as fish and jellyfish. High food quantity ensures sufficient energy intake, while high food quality, particularly in terms of FA composition, is essential for optimal growth, reproduction, and survival of these predators.

These interactions underscore the complex and dynamic nature of aquatic ecosystems, where changes at the base of the food web can cascade through higher trophic levels. Understanding these intricate relationships is vital for predicting the impacts of environmental changes on aquatic biodiversity and for managing fisheries and conservation efforts in the face of global climate change. Consequently, further investigation into the turnover time of essential biomolecules, including the dynamics of LC‐PUFA within food webs, is imperative to elucidate the fitness trajectories in key life stages and the ensuing population dynamics of predators.

## AUTHOR CONTRIBUTIONS

**Xupeng Chi:** Conceptualization (equal); data curation (equal); formal analysis (equal); methodology (equal); visualization (equal); writing – original draft (equal). **Fang Zhang:** Funding acquisition (equal); investigation (equal); methodology (equal); project administration (equal); resources (equal); supervision (equal); writing – review and editing (equal). **Song Sun:** Funding acquisition (equal); methodology (equal); project administration (equal); resources (equal); supervision (equal); writing – review and editing (equal).

## CONFLICT OF INTEREST STATEMENT

None declared.

## STATEMENT ON INCLUSION

All authors participated in the initial stages of research and study design to ensure the incorporation of diverse perspectives they represent from the outset. Where appropriate, literature authored by scientists from the region was referenced.

## Data Availability

The datasets presented in this study can be found in the online data repositories: https://figshare.com/s/bc33cdf92b149fb9e4b2.
